# SWI/SNF complexes in hematological malignancies: biological implications and therapeutic opportunities

**DOI:** 10.1186/s12943-023-01736-8

**Published:** 2023-02-21

**Authors:** Alvaro Andrades, Paola Peinado, Juan Carlos Alvarez-Perez, Juan Sanjuan-Hidalgo, Daniel J. García, Alberto M. Arenas, Ana M. Matia-González, Pedro P. Medina

**Affiliations:** 1 Department of Biochemistry and Molecular Biology I. Faculty of Sciences, University of Granada, Granada, Spain; 2 GENYO, Centre for Genomics and Oncological Research: Pfizer/University of Granada/Andalusian Regional Government, PTS Granada, Granada, Spain; 3 Instituto de Investigación Biosanitaria de Granada (ibs.GRANADA), Granada, Spain; 4 Present Address: The Francis Crick Institute, London, UK; 5 Department of Biochemistry and Molecular Biology III and Immunology, University of Granada, Granada, Spain

**Keywords:** SWI/SNF, BAF complexes, Chromatin remodeling, Epigenetics, Lymphoma, Leukemia, Multiple myeloma, Synthetic lethality, Drug resistance

## Abstract

Hematological malignancies are a highly heterogeneous group of diseases with varied molecular and phenotypical characteristics. SWI/SNF (SWItch/Sucrose Non-Fermentable) chromatin remodeling complexes play significant roles in the regulation of gene expression, being essential for processes such as cell maintenance and differentiation in hematopoietic stem cells. Furthermore, alterations in SWI/SNF complex subunits, especially in ARID1A/1B/2, SMARCA2/4, and BCL7A, are highly recurrent across a wide variety of lymphoid and myeloid malignancies. Most genetic alterations cause a loss of function of the subunit, suggesting a tumor suppressor role. However, SWI/SNF subunits can also be required for tumor maintenance or even play an oncogenic role in certain disease contexts. The recurrent alterations of SWI/SNF subunits highlight not only the biological relevance of SWI/SNF complexes in hematological malignancies but also their clinical potential. In particular, increasing evidence has shown that mutations in SWI/SNF complex subunits confer resistance to several antineoplastic agents routinely used for the treatment of hematological malignancies. Furthermore, mutations in SWI/SNF subunits often create synthetic lethality relationships with other SWI/SNF or non-SWI/SNF proteins that could be exploited therapeutically. In conclusion, SWI/SNF complexes are recurrently altered in hematological malignancies and some SWI/SNF subunits may be essential for tumor maintenance. These alterations, as well as their synthetic lethal relationships with SWI/SNF and non-SWI/SNF proteins, may be pharmacologically exploited for the treatment of diverse hematological cancers.

## Background

Hematological malignancies are cancers that arise in blood-forming tissue. In 2020, over 1.2 million people worldwide were diagnosed with hematological malignancies and over 700 thousand people died from them [1]. Taken together, hematological malignancies accounted for approximately 7.5% of new cancer diagnoses and 7.8% of cancer deaths worldwide in the year 2020, and they ranked fifth among all cancers in terms of prevalence and mortality.

Hematological malignancies are broadly classified as lymphomas, which originate in the lymphatic system; multiple myeloma (MM), which involves plasma cells from the bone marrow; and leukemias, which affect cells from the bone marrow or blood. Moreover, hematological malignancies can affect various types of cells from the blood and from the immune system at various stages of differentiation. In particular, they most commonly affect the B cell lineage (~ 67% of all hematological malignancies), followed by myeloid cells (~ 30%), and T cells or natural killer (NK) cells (~ 3%) [2]. More specific classifications of hematological malignancies can be highly complex, involving over 70 subtypes [3–5]. Among lymphomas, the most common subtypes include diffuse large B-cell lymphoma (DLBCL, ~ 15% of all hematological malignancies), marginal zone lymphoma (MZL, ~ 7%), follicular lymphoma (FL, ~ 6%), and Hodgkin lymphoma (HL, ~ 5%), all of which originate in B cells [2]. In addition, MM encompasses ~ 12% of all hematological malignancies. Among leukemias, chronic lymphocytic leukemia (CLL) represents ~ 13% of all hematological malignancies, acute myeloid leukemia (AML) represents ~ 7%, and acute lymphoblastic leukemia (ALL) represents ~ 2%. Other frequent hematological malignancies include chronic myeloproliferative neoplasms (MPN, ~ 10%) and myelodysplastic syndromes (MDS, ~ 6%), both of which affect myeloid cells in the bone marrow. Furthermore, each type of hematological malignancy can be highly heterogeneous at a molecular level and at a clinical level, and genetics-based subclassifications have been gaining popularity in recent years to better explain this heterogeneity [6–9].

This wide range of hematological malignancies arises from an aberrant regulation of hematopoiesis, a process that requires an exquisite modulation of gene expression. Different mechanisms tightly regulate hematopoiesis, among which epigenetic processes have stood out as crucial players in determining hematopoietic cell fate decisions [10, 11]. As a result, epigenetic abnormalities are frequent in hematological malignancies. Specifically, proteins involved in the modification of chromatin landscapes, such as those from SWI/SNF (SWItch/Sucrose Non-Fermentable) complexes, polycomb repressive complexes, and DNA methyltransferases, are recurrently mutated in a wide variety of hematological neoplasms [12–22]. Moreover, due to the reversible nature of epigenetics, several therapies target different epigenetic regulators to reset the altered transcriptional programs of hematological cancers and hinder tumor progression (reviewed in [23–26]). Importantly, hematological malignancies are more vulnerable to this type of therapeutic intervention than other tumors [22]. Indeed, there is increasing evidence of dependencies of hematological tumors on certain epigenetic modulators, such as chromatin remodeling complexes, highlighting the attractive potential of epigenetic therapies for the treatment of hematological malignancies.

This review summarizes the biological and therapeutic implications of SWI/SNF chromatin remodeling complexes in hematological malignancies. First, it introduces the role of SWI/SNF complexes in normal hematopoiesis. Next, it discusses the role of individual SWI/SNF subunits in the promotion, suppression, or maintenance of various hematological malignancies. Then, it overviews the recurrence of genetic, epigenetic, and gene expression alterations in SWI/SNF complex subunits in hematological malignancies. Finally, it outlines the clinical implications of SWI/SNF complexes in hematological malignancies in the contexts of SWI/SNF status as a predictor of drug response, SWI/SNF proteins as drug targets, and synthetic lethality involving SWI/SNF proteins.

## SWI/SNF complexes and their role in hematopoiesis

SWI/SNF complexes are ATP-dependent chromatin remodelers that use the energy from the hydrolysis of ATP to mobilize nucleosomes along DNA, eject them, or even change their composition [27, 28]. Consequently, these multi-subunit complexes modulate chromatin accessibility to relevant molecular players such as the transcriptional machinery, DNA-binding proteins, cofactors, regulators, and other proteins of the DNA replication and DNA repair processes [29–31]. Hence, an adequate SWI/SNF function is critical for several differentiation processes, including hematopoiesis and hematopoietic stem cell (HSC) maintenance [32–34]. However, SWI/SNF complexes may play opposing roles in normal and in malignant hematopoiesis, adding a layer of complexity to their function [35–37].

To date, 29 genes have been identified to code for SWI/SNF members, but only 12 to 15 of them coexist in a single SWI/SNF complex. Thus, there is a high variability of SWI/SNF assemblies that include lineage-restricted subunits [28, 38]. Importantly, this combinatorial potential provides a remarkable diversity of SWI/SNF complexes that are tissue-specific and contribute to controlling the transcription of lineage-specific genes [39].

In general, mammalian SWI/SNF complexes belong to three subfamilies: BRG1/BRM-associated factor (BAF), polybromo-associated BAF (PBAF), and a recently discovered subfamily named non-canonical BAF (ncBAF) [40]. The three subfamilies share a core of scaffold subunits (SMARCC1/2, and SMARCD1/D2/D3), and either of the two mutually exclusive ATPase-helicase subunits: SMARCA4 (also known as BRG1) or SMARCA2 (BRM). Additionally, each of those subfamilies contains a specific set of subunits that characterize the resulting assembly, such as the ARID (AT-rich interaction domain) subunits ARID1A/B or ARID2, and the novel non-canonical subunits BRD9, BICRA (GLTSCR1), and BICRAL (GLTSCR1L) [40, 41]. Within each SWI/SNF subfamily, the subunits can be arranged in different combinations. The resulting assemblies are key to determine not only the genomic sites of action of the different SWI/SNF complexes, but also their interaction with other transcription factors [42–45], DNA repair proteins [31, 46], and DNA replication proteins [47, 48], among many others. Increasing evidence has demonstrated that these changes in the SWI/SNF interactome are crucial for the maintenance of hematological gene expression patterns. Specifically, SWI/SNF complexes can also interact with hematopoietic-specific transcription factors, including EKLF, RUNX1, PU.1, IKAROS, GATA1, and CEBPα [49–53].

Growing evidence has demonstrated the essential role of SWI/SNF complexes in normal hematopoiesis (Table 1). Independent studies have described the important functions of many SWI/SNF subunits, such as ACTL6A, ARID1A, ARID2, PBRM1, PHF10, and SMARCA2 for the maintenance of HSCs [37, 54–58]. In addition, some SWI/SNF subunits play relevant roles in both myeloid and lymphoid differentiation, such as ARID1A [55, 59] and SMARCA4 [60–67]. On the other hand, other subunits are specific to certain processes within one of those lineages. For instance, SMARCE1 is involved in the differentiation of CD4+ and CD8+ T cells [64], while ARID1B and SMARCD2 regulate erythropoiesis [68] and granulocytic maturation [69, 70], respectively.

**Table 1:** Hematological processes regulated by the mammalian SWI/SNF complex

Subunit	Biological process	Refs.
ACTL6A	Maintenance of hematopoietic stem cells (HSCs)	[54]
ARID1A	Maintenance of HSCs and differentiation of both myeloid and lymphoid lineages	[55]
	T cell development	[59]
ARID1B	Erythropoiesis	[68]
ARID2	Maintenance of HSCs and erythropoiesis	[37]
	Regeneration of lymphoid lineage	[35]
BCL11A	B-cell and T-cell development	[71]
	Regulation of ɣ-globin gene	[72–75]
	Maintenance of HSCs	[76]
BCL11B	Thymocyte differentiation	[77]
BRD9	Functional modulation of Regulatory T (T-reg) cells	[78]
PBRM1	Maintenance of HSCs	[56]
PHF10	Maintenance of HSCs and myeloid lineage development	[57]
SMARCA2	Regulatory function in HSCs and HPCs (hematopoietic progenitor cells)	[58]
SMARCA4	Erythroid development	[60–62]
	Granulocytic maturation	[63]
	T-cell differentiation	[64–66]
	Development of pro-B cells	[67]
	Formation and maintenance of HPCs	[79]
	Myeloid lineage differentiation	[80]
SMARCC1	Development of pro-B cells	[67]
	Thymocyte maturation	[81]
SMARCD2	Granulocytic maturation	[69, 70]
SMARCE1	T-cell differentiation	[64]

## Contribution of SWI/SNF complexes to hematological malignancies

Because SWI/SNF complexes are involved in the homeostasis of hematological processes, alterations in SWI/SNF subunits can contribute to the onset or progression of hematological malignancies. The roles of different SWI/SNF subunits in different hematological malignancies can be highly diverse, ranging from being tumor suppressive, to being required for tumor maintenance, or even being oncogenic (Table 2). In line with this functional diversity, as will be detailed later, genetic alterations in SWI/SNF subunits are highly recurrent in various hematological malignancies, such as DLBCL [7, 8, 82, 83], whereas they are extremely rare in others, such as AML (excluding the acute promyelocytic leukemia (APL) subtype) [51, 84]. When present, SWI/SNF mutations in hematological malignancies usually cause loss of function, suggesting a tumor suppressor role. On the other hand, in certain contexts, such as in non-APL AML, normal function of at least some SWI/SNF subunits may be required for tumor maintenance [36, 51, 85]. However, although the essentiality of SWI/SNF for non-APL AML maintenance has been extensively demonstrated in vivo, to our knowledge few in vivo studies have explored the functional consequences of SWI/SNF alterations in other hematological malignancies [86].

**Table 2:** SWI/SNF role in hematological tumors

	Subunit	Role (TS/OG/TM)^a^	Malignancy	Described effect	Refs.
ATPase-Helicase	SMARCA4	TM	AML	Maintenance of leukemic cells	[51, 85, 87]
	SMARCA2/4	TM	AML	Higher dependency on SMARCA4 and SMARCA2 in leukemic cells	[88]
ARID subunits	ARID1A	TM	AML	Maintenance of leukemic cells	[51]
		TS	AML	Its loss increases cell proliferation and inhibits apoptosis of AML cells	[89]
	ARID1B	TS	AML	Its loss accelerates leukemogenesis and promotes leukemic maintenance	[36]
		TS	APL	Required for cellular differentiation	[84]
	ARID2	TS	AML	Its loss accelerates leukemogenesis	[36, 37]
		TM	AML	Maintenance of leukemic cells	[36]
Core subunits	SMARCB1	TS	–	Its loss promotes lymphomagenesis (CD8^+^ T cells)	[90]
		TS	CML	Its loss accelerates leukemogenesis	[91]
		TS	AML	Its loss activates oncogenic gene expression programs	[92]
	SMARCC1	TM	AML	Maintenance of leukemic cells	[51]
	SMARCD1	TM	AML	Maintenance of leukemic cells	[51]
	SMARCD2	TM	AML	Maintenance of leukemic cells	[51, 85]
Accessory subunits	ACTL6A	TM	AML	Maintenance of leukemic cells	[51]
	BCL7A	TS	DLBCL	Impairs proliferation of DLBCL cells	[86]
	BCL11A	OG	CLL, NHL, and HL	Chromosomal translocations or amplifications of this gene are found in lymphoid malignancies	[93]
		OG	AML	Acceleration of the onset of Trib1-induced AML. Its loss reduces leukemogenicity	[94]
		OG	–	Increases the onset of leukemia of either myeloid or lymphoid lineage	[95]
		TM	NKTL	Its loss increases apoptosis and reduces proliferation of NKTL cells	[96]
	BCL11B	TS	CML	Haploinsufficiency of *Bcl11b* promotes oncogenicity of the *Bcr-Abl* fusion in CML	[97]
		TS	T-ALL	Haploinsufficient tumor suppressor	[98]
		OG	ALAL	Cell self-renewal, myeloid differentiation blockage, activation of T cell genes	[99, 100]
	DPF2	TM	AML	Maintenance of leukemic cells	[85]
	BRD9	TM	ALL, MM	Its loss promotes apoptosis and sensitizes ALL and MM cells to chemotherapy.	[101]
		TM	AML	Maintenance of leukemic cells	[102]

^a^*TS* Tumor suppressor, *OG* Oncogene, *TM* Tumor-Maintenance, *ALAL* acute leukemia of ambiguous lineage, *ALL* acute lymphoblastic leukemia, *AML* acute myeloid leukaemia, *APL* acute promyelocytic leukaemia, *CLL* chronic lymphocytic leukemia, *CML* chronic myeloid leukaemia, *DLBCL* diffuse large B cell lymphoma, *HL* Hodgkin lymphoma, *MM* multiple myeloma, *NKTL* natural killer T cell lymphoma, *NHL* non-hodgkin lymphoma; -: unclassified

Importantly, genetic alterations involving different SWI/SNF complex subunits in the same disease can have vastly different functional effects because the remaining residual SWI/SNF complexes may have different biological activities [36]. For instance, the oncogenic fusion protein KMT2A-MLLT1 (also known as MLL1-ENL1), which has been recurrently observed in ALL, AML, and MLL (mixed-lineage leukemia) [103], forms aberrant SWI/SNF complexes named EBAFb (ENL-associated BAF-containing BAF250b) [104]. These EBAFb complexes, which contain SMARCA4 as their catalytic subunit, activate the transcription of genes that are aberrantly expressed in MLL [104]. In the remainder of this section, we summarize the main functional studies on the role of SWI/SNF subunits in hematological malignancies, most of which have been performed in myeloid leukemia.

### ATPase-helicases: SMARCA4 and SMARCA2

Although, in many tumor types, *SMARCA4* displays loss-of-function mutations characteristic of a tumor suppressor, an additional role as a tumor-supportive gene in certain malignancies is emerging. Indeed, SMARCA4-containing SWI/SNF complexes may be essential for the maintenance of AML, opening new therapeutic possibilities [51, 59, 85, 102]. Mechanistically, SMARCA4-containing SWI/SNF complexes may modulate the well-known oncogene *MYC* specifically in AML. This modulation has been thoroughly demonstrated in the mouse cell line RN2 (*KMT2A*-rearranged *NRAS*^*G12D*^ AML) [51, 102], as well as in the human cell line ME-1 (AML harboring the *CBFB-MYH11* fusion gene) [105]. However, another study in a different murine AML model (*KMT2A*-rearranged *NRAS*^*WT*^) failed to reproduce the modulation of *Myc* by SMARCA4, although it did confirm that SMARCA4, along with SMARCD2 and DPF2, are required for AML maintenance [85].

Regarding SMARCA2, its individual role in hematological malignancies remains unknown. In one study, dual loss of SMARCA2 and SMARCA4 activity either by using allosteric dual inhibitors, such as BRM011 and BRM014, or by knock-down approaches, significantly impaired the viability of a diverse panel of AML cell lines, including those that did not display sensitivity to *SMARCA4* knockdown [88].

### ARID subunits: ARID1A, ARID1B, ARID2

The ARID subunits of SWI/SNF complexes can show both tumor suppressor and tumor supportive properties depending on the cellular model, disease subtype, and disease stage in which they are evaluated. For example, inhibition of *Arid1a* abrogated proliferation in RN2 cells, suggesting that ARID1A-containing SWI/SNF complexes promoted AML maintenance in this context [51]. However, another report indicated that ARID1A may act as a barrier to uncontrolled cell division in two human AML cell lines: HL-60 (APL) and THP-1 (*KMT2A*-rearranged) [89]. In particular, decreasing ARID1A expression in both cell lines suppressed apoptosis and boosted the proliferating ability of AML cells through the TGF-ꞵ1/SMAD3 pathway.

Regarding ARID1B, despite it sharing high homology with ARID1A [106], this subunit has been more linked to a suppressive role in leukemia rather than to a contributing role in tumor maintenance. Specifically, in a recent report by Bluemn et al., knockout of *Arid1b* promoted both AML initiation and progression in a mouse model of *KMT2A*-rearranged AML [36]. Interestingly, the authors failed to find phenotypical consequences upon knocking down *Arid1b* in vitro. In addition, another study showed that knocking down *ARID1B* in an APL cell line (NB4) under treatment with All-trans retinoic acid (ATRA) blocked blast differentiation [84].

In contrast to ARID1B, the effects of ARID2 may be dependent on the AML stage. In particular, Bluemn et al. found that loss of *Arid2* enhances leukemogenesis at the initial stages of AML, but a functioning ARID2 is later required for AML maintenance [36]. Similarly to *Arid1b*, the phenotypical effects of knocking out *Arid2* were observed in vivo but not in vitro. Moreover, the transcriptomic changes were different and, in some cases, opposing when comparing the effects of knocking out *Arid1b* or *Arid2* in the same mouse model. A likely explanation for this discrepancy is that, since ARID1B and ARID2 belong to different SWI/SNF subfamilies (BAF and PBAF, respectively), the biological activity of the residual complexes remaining upon knockout of *Arid1b* or *Arid2* may be different.

### Core subunits: SMARCB1, SMARCC1, SMARCD1/2

The core subunits of SWI/SNF complexes have been described to play essential roles in different hematological malignancies because of their crucial function as structural scaffolds. For instance, the knockdown of *Smarcc1*, *Smarcd1*, or *Smarcd2* impaired the survival of mouse AML cells, confirming their tumor-maintaining role in this tumor type [51, 85]. Moreover, the loss of *Smarcb1* can also induce the development of T-cell lymphoma in mouse models [90]. Regarding the latter core subunit, its absence in AML cells generates a residual SWI/SNF complex with SMARCC1 as one of its core subunits, which regulates the expression of oncogenic programs crucial for the survival and migration of AML cells [92].

### Accessory subunits

The specific role of some accessory SWI/SNF subunits remains largely unknown. However, their mutational frequency in certain tumor types suggests that they are important contributors to tumorigenesis. This is the case for BCL7A (B-cell CLL/lymphoma 7 protein family member A), which is expressed in the nuclei of germinal center B lymphocytes, but it is lost in mature plasma cells. This subunit is frequently mutated in DLBCL, and reintroduction of wild type BCL7A impaired the proliferation of DLBCL cells both in vitro and in vivo, pointing to a tumor suppressor role [86]. Moreover, recurrent mutations in its first splice donor site cause a deletion of 27 amino acids in the N-terminal portion of the protein, preventing its assembly into the SWI/SNF complex [86].

On the other hand, the accessory subunit BCL11A may have oncogenic functions across multiple hematological malignancies in view of its genetic patterns and expression alterations, which will be detailed in later sections. BCL11A is a zinc-finger transcription factor highly expressed in several hematopoietic lineages. It has been observed that BCL11A may induce the cell cycle progression of hematopoietic cells by directly inhibiting p21 expression [95], and it may deregulate the expression of a plethora of genes involved in apoptosis [107] or those regulated by other myeloid tumor suppressors such as PU.1 [94].

The paralog of BCL11A, BCL11B, is required for T cell development [77]. As a result, it can have either tumor suppressive or oncogenic functions in different hematological malignancies. In T-ALL, *BCL11B* may be a haploinsufficient tumor suppressor gene, in part because it may help maintain a differentiated T cell state [98]. Interestingly, a haploinsufficient tumor suppressor role of *BCL11B* has also been proposed in CML [97]. In contrast, in a subset of acute leukemias of ambiguous lineage (ALALs), *BCL11B* acts as an oncogene by driving specific gene expression programs [99, 100]. Mechanistically, it has been proposed that these leukemias originate when *BCL11B* becomes aberrantly activated in hematopoietic stem and progenitor cells (HSPCs), which promotes cell self-renewal, blocks myeloid differentiation, and activates the transcription of T lineage genes without proper differentiation into T cells, giving rise to the ambiguous lineage phenotype [99].

Finally, the bromodomain-containing subunit BRD9 may be essential for the maintenance of various hematological malignancies, including ALL, MM, and AML [101, 102]. In the mouse AML cell line RN2, BRD9 associates with SMARCA4-containing SWI/SNF complexes [102]. Knockdown of BRD9 impaired cell growth in RN2 cells and in a panel of human leukemia cell lines, but not in human epithelial cell lines or in mouse embryonic fibroblasts. This lineage-specific dependence highlights BRD9 as a potential target for novel chemotherapeutic agents, as will be detailed in later sections.

## Genetic alterations of SWI/SNF subunits in hematological malignancies

Mutations in epigenetic modifiers and chromatin remodeling genes, including SWI/SNF complexes, are a major hallmark of many hematological malignancies, such as various NHLs [108], T cell lymphomas [109, 110], and dendritic cell neoplasms [111]. In addition, even in those hematological malignancies in which SWI/SNF alterations are not a major hallmark, inactivating mutations in SWI/SNF genes, especially *ARID1A*, are usually present at low frequencies (≤ 5%) [112, 113].

Various SWI/SNF genes, such as *ARID1A/1B/2* and *SMARCA2/4*, are recurrently mutated across a wide variety of hematological and non-hematological cancers, whereas others, such as *BCL7A*, are specifically mutated in certain hematological malignancies [86, 114, 115]. Although the molecular mechanisms by which SWI/SNF mutations may promote such a wide variety of cancers remain largely unknown, two main models have been proposed (reviewed in [116]). One model proposes that, because SWI/SNF mutations impair DNA repair and genome stability, they create an overall mutagenic environment that may be favorable in different cancer contexts. This model is supported by the observation that SWI/SNF deficient tumors often have a high mutation burden [117], but it is unclear if the high mutation rate of SWI/SNF is the cause or the consequence of the high tumor mutation burden. Alternatively, another model suggests that SWI/SNF mutations affect the genome-wide chromatin accessibility patterns, possibly disrupting the function of lineage-specific transcription factors, which may further alter transcriptional programs in a cell type-specific manner. Indeed, as discussed in previous sections, many SWI/SNF subunits (including the widely mutated *ARID1A* and *SMARCA4*) play specific roles in the differentiation of lymphoid and myeloid lineages and in the maintenance of some hematological malignancies. Overall, further research should clarify the relative contribution of each proposed mechanism in different cancer contexts.

Several mechanistic studies support the idea that *SMARCA4* mutations can promote cell type-specific oncogenesis by dysregulating lineage-specific transcriptional programs. Across a wide variety of hematological and non-hematological cancers, mutations in *SMARCA4* are usually missense and heterozygous, and many of them have been proven to be dominant negative in at least some cellular contexts [118, 119]. *SMARCA4* missense mutations frequently disrupt conserved positions involved in either its ATPase activity, its DNA helicase activity, or its overall tridimensional structure. Interestingly, in *Smarca4-*knockout mouse embryonic stem cells, which also lack expression of *Smarca2*, overexpression of human *SMARCA4* harboring different missense mutations in various conserved positions had converging effects on chromatin accessibility [118]. In particular, the tested mutations mostly decreased the accessibility of a large number of enhancers and superenhancers, many of which were specific to either pro-B cells, T cells, macrophages, bone marrow tissue, or thymus, which could explain why *SMARCA4* mutations are ubiquitous across different cancer types while promoting cell type-specific oncogenesis. For example, in a previous study, SMARCA4 had been reported to promote the expression of the *MYC* oncogene specifically in leukemia, even though both *MYC* and *SMARCA4* are expressed ubiquitously across many cell types [51]. To explain this phenomenon, it was proposed that SWI/SNF binds to a *MYC* superenhancer only in leukemia cells, and that the superenhancer is bound by lineage-specific transcription factors. Of note, these findings were not reproduced by a later study in a different AML model [85]. In practice, the effect of mutations in *SMARCA4* in cancer must be interpreted in the context of the status of its paralog *SMARCA2*. Indeed, in *SMARCA4*-deficient lung cancer cell lines, knocking down *SMARCA2* decreased cell viability, and subsequently restoring different *SMARCA4* mutants had varying abilities to compensate for the phenotype caused by *SMARCA2* loss [119]. This phenomenon has critical implications in the context of anti-SMARCA2/4 therapeutic interventions, as the status of one protein may determine the efficacy of inhibiting the other. Finally, intriguingly, some *SMARCA4* mutants that had negligible chromatin remodeling activity were able to revert the growth defects caused by *SMARCA2* depletion [119].

Regarding *ARID1A*, about half of its mutations found across different hematological and non-hematological cancers are truncating (mainly nonsense or frameshift) [118]. Whereas truncating mutations in *ARID1A* are under strong positive selection, missense mutations have unknown significance and they have been proposed to be passengers [120]. In mice, loss of *Arid1a* impairs the ability of hematopoietic stem cells to differentiate into myeloid and lymphoid lineages by decreasing the accessibility of key loci that regulate hematopoiesis, such as *Cebpa*, *Cd34*, *Csf1*, *IL6ra*, and *Gata2* [55]. Therefore, this observation may explain why loss-of-function mutations in *ARID1A* are observed in a wide variety of hematological cancers.

In contrast to *SMARCA2/4* and *ARID1A/1B/2* genes, *BCL7A* seems to be specifically mutated in lymphomas that are either derived from or phenotypically similar to germinal center B cells, such as germinal center B cell-like (GCB) DLBCL [86], FL [121], Burkitt lymphoma (BL) [122], and MM [123]. In all cases, *BCL7A* mutations accumulate in its promoter, its 5’-untranslated region (UTR), its first exon, and its first intron, following mutational patterns consistent with aberrant somatic hypermutation (aSHM) caused by off-target activity of the activation-induced cytidine deaminase (AID) enzyme. AID activity occurs during the germinal center reaction, and therefore AID mutational signatures are specific to germinal center-derived malignancies [124]. In DLBCL, certain ‘hotspot’ *BCL7A* mutations, such as those at its first splice donor site, may be bona fide driver events [86]. In contrast, it is currently unclear if *BCL7A* mutations are under selection in FL, BL, and MM. Finally, the hypermutated region of *BCL7A* has been identified as a super-enhancer in DLBCL [125].

Another recurrent alteration involving SWI/SNF genes across various hematological malignancies is the gain or amplification of chromosome 2p16.1, which affects both *BCL11A* and the nearby *REL* gene, and which can lead to *BCL11A* overexpression in some cases [93, 126]. Gain of chromosome 2p16.1 has been recurrently observed in DLBCL [7, 8], FL [108], BL [108], HL [127], and primary mediastinal B-cell lymphoma (PMBL) [128, 129]. However, while some reports state that *BCL11A* is the main specific target of the copy number gains [108], others have found that the nearby *REL* gene is amplified at a greater degree than *BCL11A* [129, 130], and many argue that *BCL11A* is not a specific target of the recurrent copy number gains [8, 127, 131].

**Fig. 1:**
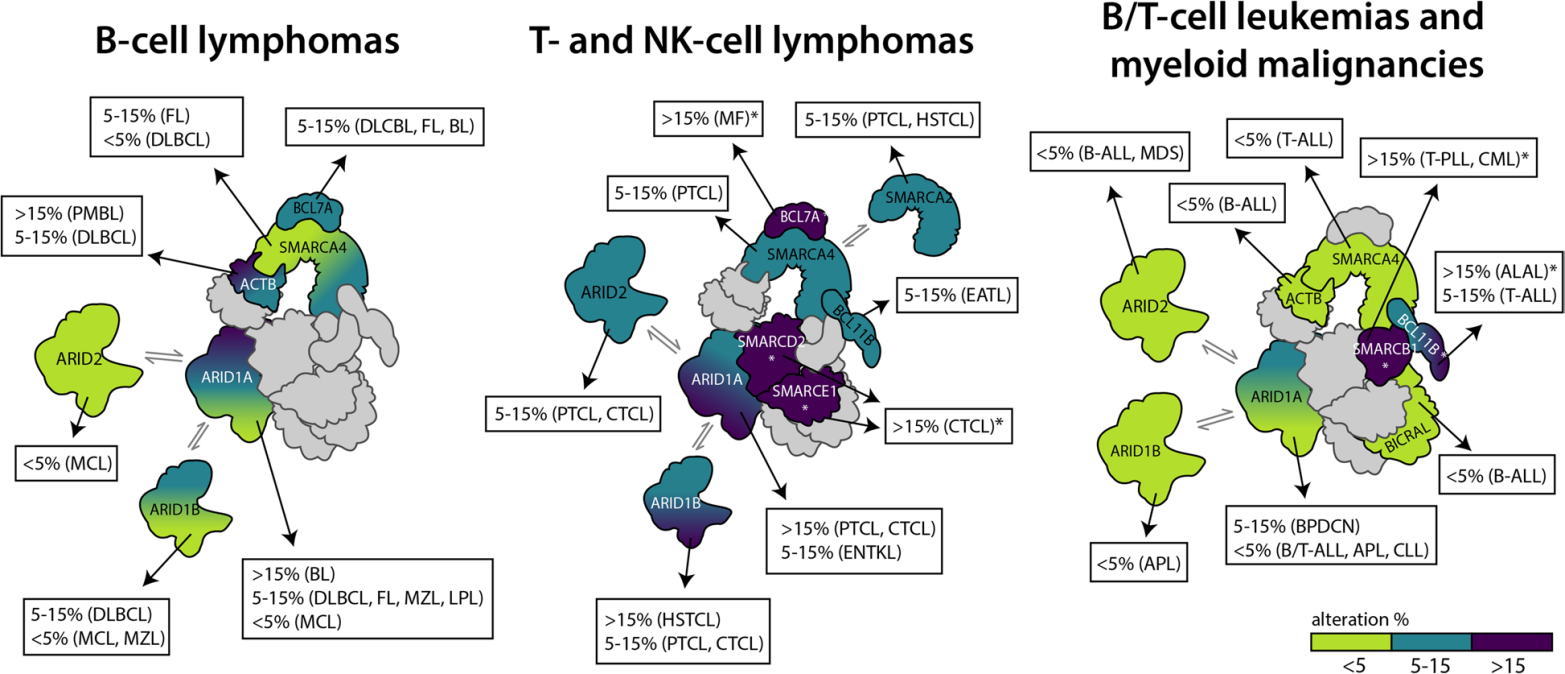
Summary of the main genetic alterations in SWI/SNF subunits in hematological malignancies. Because alteration frequencies can vary greatly between cohorts of the same disease (Table 3), they have been roughly categorized in three discrete groups. Double arrows indicate paralogous subunits that can play equivalent roles in different complexes. ALAL: acute leukemia of ambiguous lineage; ALL: acute lymphoblastic leukemia; APL: acute promyelocytic leukemia; BL: Burkitt lymphoma; BPDCN: blastic plasmacytoid dendritic cell neoplasm; CLL: chronic lymphocytic leukemia; CML: chronic myeloid leukemia; CTCL: cutaneous T cell lymphoma; DLBCL: diffuse large B cell lymphoma; EATL: enteropathy-associated T cell lymphoma; ENKTL: extranodal NK/T cell lymphoma; FL: follicular lymphoma; HSTCL: hepatosplenic T cell lymphoma; LPL: Lymphoplasmacytic lymphoma; MCL: mantle cell lymphoma; MDS: myelodysplastic syndrome; MF: mycosis fungoides; MZL: marginal zone lymphoma; PLL: prolymphocytic leukemia; PMBL: primary mediastinal large B-cell lymphoma; PTCL: peripheral T cell lymphoma, not otherwise specified. *Limited study, see Table 3

In the remainder of this section, the genetic alterations (point mutations, copy number alterations, and genomic rearrangements) reported in SWI/SNF genes in hematological malignancies of various cell origins will be summarized (Table 3 and Fig. 1). Results from Data Release 33.1 of the International Cancer Genome Consortium (ICGC) and Data Release 12.0 of the Genomics, Evidence, Neoplasia, Information, Exchange (GENIE) Project, as well as individual (often disease-specific) studies, will be included [112, 113]. Whenever appropriate, data across multiple studies will be integrated but, when an individual study differs greatly from other work, it will be reported separately. Such differences between studies may stem from various factors: (i) small sample sizes, which can lead to inaccurate estimates of mutation frequencies; (ii) differences in the composition of the cohorts, for example in terms of disease subtypes; (iii) sequencing strategies (for example, targeted sequencing may not cover all SWI/SNF genes); (iv) differences in data processing and filtering of mutations; and (v) inclusion of matched normal samples. Indeed, studies that report unusually high mutation frequencies of individual genes often lack matched normal samples.

**Table 3:** Genetic alterations in SWI/SNF complex genes in hematological malignancies

Category	Malignancy	Gene	Alteration	Freq.	References
B cell lymphomas	DLBCL	*ARID1A*	Mut.	7-9%	[7–9, 82, 83, 108, 112, 130, 132]
		*ARID1B*	Mut.	7%	[7–9, 82, 83, 108, 112, 130, 132]
		*BCL7A*	Mut.	6-12%	[7–9, 82, 83, 108, 112, 130, 132]
		*ACTB*	Mut.	5-10%	[7–9, 82, 83, 108, 112, 130, 132]
		*SMARCA4*	Mut.	1-5%	[7–9, 82, 83, 108, 112, 130, 132]
	FL	*ARID1A*	Mut.	6-15%	[108, 112, 133, 134]
		*SMARCA4*	Mut.	5-8%	[108, 112, 133, 134]
		*BCL7A*	Mut.	4-19%	[108, 112, 133, 134]
	BL	*ARID1A*	Mut.	15-45%	[112, 122, 135, 136]
		*SMARCA4*	Mut.	14-38%	[108, 112, 122, 135–138]
		*BCL7A*	Mut.	7%	[108, 138]
	HL	*ARID1A*	Mut.	9-26%^b^	[112, 139, 140]
		*ACTB*	Mut.	26%^b^	[140]
	PMBL	*ACTB*	Mut.	33%	[141]
	MCL	*SMARCA4*	Mut.	6-10%	[108, 112, 142, 143]
		*ARID1A*	Mut.	3-6%	[108, 112, 142, 143]
		*ARID1B*	Mut.	3-6%	[108, 112, 142, 143]
		*ARID2*	Mut.	3-6%	[108, 112, 142, 143]
	MZL	*ARID1A*	Mut.	7%	[112, 144–146]
		*ARID1B*	Mut.	4%	[112, 144, 145]
	LPL	*ARID1A*	Mut.	5-17%	[112, 147, 148]
	LPL: WM	*ARID1B*	Del.	50%^b^	[148]
T and NK cell lymphomas	PTCL, NOS	*ARID1A*	Mut. + Del.	8-25%	[110, 112, 149]
		*ARID2*	Mut. + Del.	2-14%	[110, 112, 149–151]
		*ARID1B*	Mut. + Del.	4-11%	[110, 149–151]
		*SMARCA2*	Mut. + Del.	19%^b^	[149]
		*SMARCA4*	Mut. + Del.	8%^b^	[149]
		*SMARCA4*	Amp.	19%^a,b^	[149]
	CTCL	*ARID1A*	Del.	28-58%	[152–155]
		*ARID1A*	Mut.	8%	[112, 152–157]
		*SMARCE1*	Amp.	20%^b^	[153]
		*SMARCD2*	Amp.	20%^b^	[153]
		*ARID2*	Del.	8%^b^	[153]
	CTCL: MF	*BCL7A*	Del.	44%^a^	[158]
	ENKTL	*ARID1A*	Mut.	6%	[159–163]
	HSTCL	*ARID1B*	Mut.	18%	[109]
		*SMARCA2*	Mut.	10%	[109]
	EATL	*BCL11B*	Mut.	12%	[164]
B and T cell leukemias	B-ALL	*ACTB*	Mut. + Del.	< 1%	[165, 166]
		*ARID1A*	Mut. + Del	< 1%	[166]
		*ARID2*	Mut. + Del	< 5%	[165, 166]
		*BICRAL*	Mut.	< 1%	[165]
	T-ALL	*ARID1A*	Mut.	3%	[165, 166]
		*BCL11B*	Mut.	8-10%	[98, 112, 165–167]
		*SMARCA4*	Mut.	3%	[165, 166]
	T-PLL	*SMARCB1*	Del.	55%^b^	[168, 169]
	CLL	*ARID1A*	Mut.	< 5%	[112, 113]
	ALAL	*BCL11B*	Amp. + Trl.	33%^b^	[99, 100]
Myeloid / dendritic cell malignancies	APL	*ARID1A*	Mut.	5%	[84]
		*ARID1B*	Mut.	3%	[84]
	CML	*SMARCB1*	Del.	30-80%^b^	[91, 170]
	MDS	*ARID2*	Mut. + Del.	2%	[171]
		All SWI/SNF	Mut.	17.8%^a^	[172]
	BPDCN	*ARID1A*	Mut.	11%	[111, 112, 173–176]

Alteration frequencies (Freq.) are approximate estimates or ranges based on our integration of multiple studies; see main text for more details

*Amp.* amplification, *Del*. deletion, *Mut.* point mutation, *Trl.* translocation, *AITL* angioimmunoblastic T cell lymphoma, *ALAL* acute leukemia of ambiguous lineage, *ALL* acute lymphoblastic leukemia, *APL* acute promyelocytic leukemia, *BL* Burkitt lymphoma, *BPDCN* blastic plasmacytoid dendritic cell neoplasm, *CLL* chronic lymphocytic leukemia, *CML* chronic myeloid leukemia, *CTCL* cutaneous T cell lymphoma, *DLBCL* diffuse large B cell lymphoma, *EATL* enteropathy-associated T cell lymphoma, *ENKTL* extranodal NK/T cell lymphoma, *FL* follicular lymphoma, *HL* Hodgkin lymphoma, *HSTCL* hepatosplenic T cell lymphoma, *LPL* Lymphoplasmacytic lymphoma, *MCL* mantle cell lymphoma, *MDS* myelodysplastic syndrome, *MF* mycosis fungoides, *MZL* marginal zone lymphoma, *PLL *prolymphocytic leukemia, *PMBL* primary mediastinal large B-cell lymphoma, *PTCL, NOS* peripheral T cell lymphoma, not otherwise specified, *WM* Waldenström macroglobulinemia

^a^Results not reproduced by similar studies

^b^Results were obtained in cohorts of limited size (*N* < 50) and/or lacking matched normal samples, and therefore estimated percentages may be inaccurate. In the case of ALAL, it is a highly heterogeneous disease and the estimated frequency of *BCL11B* alterations may heavily depend on the patient inclusion criteria

### B-cell lymphomas

#### Diffuse large B-cell lymphoma

**Fig. 2:**
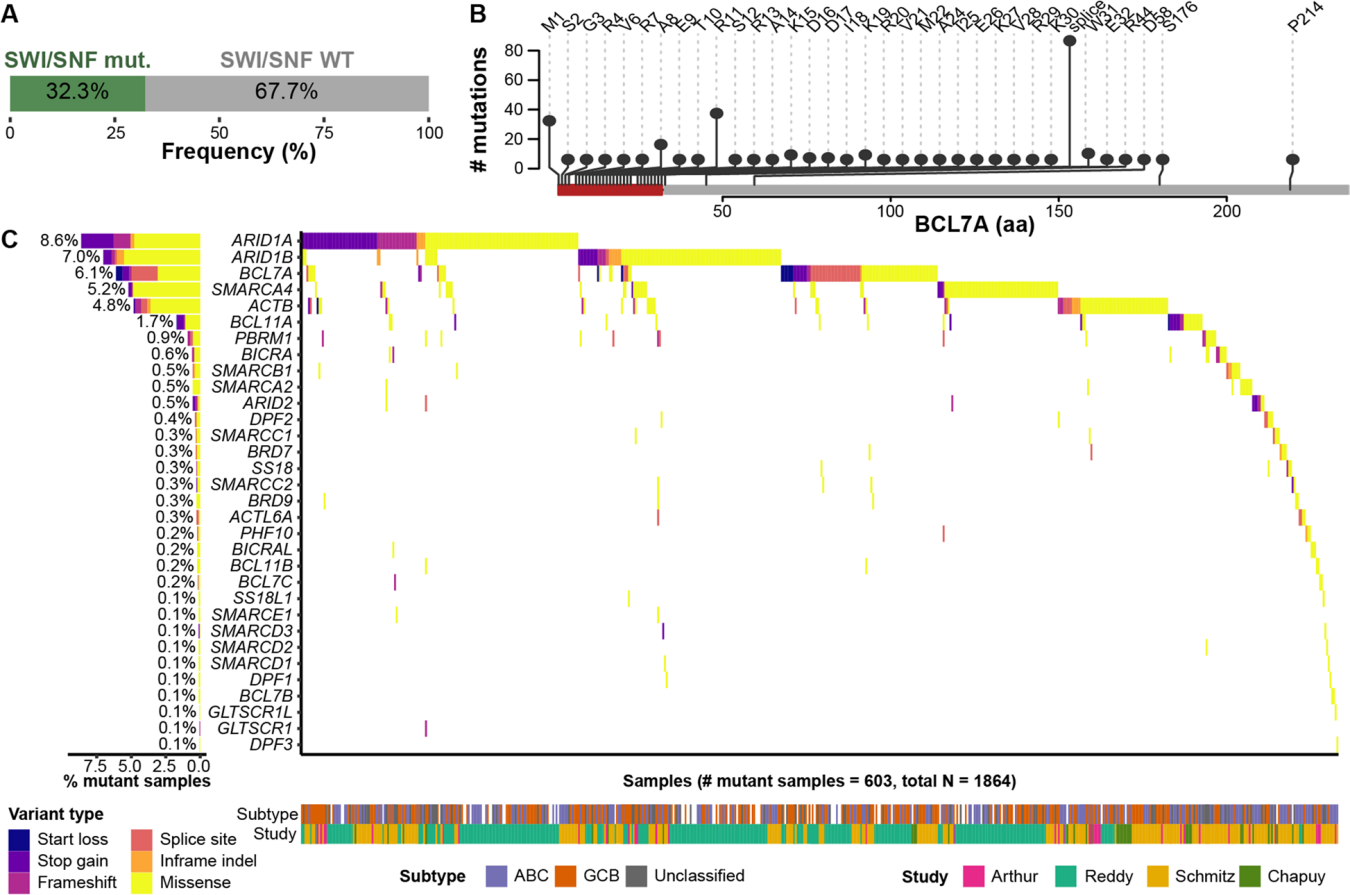
Mutations in SWI/SNF genes in diffuse large B cell lymphoma (DLBCL). **A** Proportion of DLBCLs that have at least one mutation (mut.) in one SWI/SNF gene in whole-exome sequencing (WES) or whole-genome sequencing (WGS) studies. WT: wild type. Data sources: [7, 8, 82, 83]. In the datasets of Reddy et al. and Schmitz et al., previously missed splice site mutations from our previous reports have been added [86, 115]. **B** Distribution of mutations in BCL7A, shown at the protein level. Exon 1 (amino acids 1-31) is highlighted in red. Data sources: [7–9, 82, 83, 108]. **C** Distribution of mutations in SWI/SNF genes in DLBCL in WES or WGS studies. Data sources: [7, 8, 82, 83]. For the cohort of Chapuy et al., only paired samples were included in the plot. ABC: activated B cell-like; GCB: germinal center B cell-like

DLBCL is the most common type of NHL. SWI/SNF subunits are mutated in over a third of DLBCLs (Fig. 2A). According to an integration of whole-exome sequencing (WES) and whole-genome sequencing (WGS) studies in over 2000 patients, the top mutated SWI/SNF genes in DLBCL are *ARID1A* (8.6%), *ARID1B* (7.0%), *BCL7A* (6.1%), *SMARCA4* (5.2%), and *ACTB* (4.8%) (Fig. 2C) [7, 8, 82, 83]. Moreover, targeted sequencing studies in DLBCL, collectively including over 2400 samples, have found the same five SWI/SNF genes as highly mutated at similar frequencies [9, 108, 112, 130, 132]. In addition, gains of chromosome 2p16.1, which contains *BCL11A*, affect 7-28% DLBCLs [7, 8, 108, 129, 130]. Remarkably, over a third of *ARID1A* mutations are truncating (nonsense or frameshift). Also of note, the mutation frequency of *BCL7A* and, consequently, of the SWI/SNF complex, is strongly dependent on the proportion of GCB DLBCLs in the cohort [7, 8, 82, 83].

Over 50%, and up to 70%, of mutations in *BCL7A* in DLBCL are either truncating or within three mutational hotspots: the start codon, arginine 11, or the first splice donor site (Fig. 2B) [9, 86]. These mutations often affect different alleles from the same sample, suggesting biallelic inactivation patterns that are consistent with a tumor suppressor role of *BCL7A* in DLBCL [86]. The strongest mutational hotspot in *BCL7A* in DLBCL is its first splice donor site, which had been overlooked by some large-scale studies [82, 86]. Recently, our group showed that mutations in the first splice donor site of *BCL7A* cause an in-frame deletion of 27 codons in its first exon, impairing its tumor suppressor function in DLBCL [86].

#### Follicular lymphoma

Nearly all FLs are mutated in at least one chromatin remodeling gene, and the SWI/SNF complex is among the most highly mutated chromatin remodeling complexes in FL [108, 133]. In particular, various reports in independent FL cohorts have found recurrent point mutations in *ARID1A* (mutation frequency ~ 6-15%, combined *N* = 973) and in *SMARCA4* (5-8%) [108, 112, 133, 134]. Importantly, mutations in *ARID1A* have been associated with better disease-free survival in FL [177]. Other SWI/SNF genes harboring recurrent point mutations in FL include *BCL7A*, *ARID1B*, *ARID2*, and *BCL11B* [112]. Moreover, chromosome 2p16.1 is amplified in ~ 22% FLs [108].

Recurrent *BCL7A* mutations have been consistently observed in independent FL cohorts, but the mutation frequencies are highly variable, ranging from 4% (*N* = 138) [133] to 11% (*N* = 199) [108] and up to 19% (*N* = 105) [134]. In all cases, the mutations concentrate in the first exon and the first splice donor site of *BCL7A*, as was observed in DLBCL [86]. Indeed, the mutations are thought to be caused by aSHM in late stages of the disease and they may be associated with FL transformation to DLBCL [121]. On the other hand, this model does not explain the large discrepancies in the mutation rate of *BCL7A* between cohorts, as the two most discrepant studies both focused on early FL. Overall, more work will be necessary to clarify the role of *BCL7A* mutations and aSHM in FL progression and transformation.

#### Burkitt lymphoma

More than 1 in every 4 BLs harbors a mutation in at least one SWI/SNF gene [112, 122, 135–138]. Three SWI/SNF genes are mutated in BL at frequencies above 15% in a mutually exclusive manner and have been proposed as drivers of the disease: *ARID1A*, *SMARCA4,* and *BCL7A* [135, 137, 138]. Other SWI/SNF genes recurrently mutated in BL include *ARID2*, *SMARCA2, PBRM1, BCL11A, ACTL6A*, *SMARCC2*, *SMARCD1*, *DPF1, BRD7*, and *BRD9* [112, 137]. However, all of them are mutated at frequencies below 5%, and, to our knowledge, all their mutations reported so far are missense of unknown significance. Moreover, amplification of chromosome 2p16.1 occurs in ~ 11% BLs [108].

*ARID1A* is mutated in ~ 15-45% of BL patients [112, 122, 135, 136]. Mutations in *ARID1A* are often truncating (e.g., nonsense or frameshift), causing loss of function. Interestingly, *ARID1A* has been identified as an essential gene in BL by CRISPR-knockout screening [122]. Moreover, germline mutations in *ARID1A* may predispose to BL [135].

*SMARCA4* is mutated in ~ 14-38% of BLs [108, 112, 122, 135–138]. In contrast to *ARID1A*, the vast majority of *SMARCA4* mutations in BL are missense, but still they are predicted to have a high functional impact [136]. Among SWI/SNF mutations, those in *SMARCA4* are the most enriched in BL compared to other non-Hodgkin lymphomas [108]. Furthermore, *SMARCA4* mutations co-occur with *MYC* translocations in BL [108].

*BCL7A* was initially reported as a target of a three-way chromosomal rearrangement in a BL cell line [178]. Since then, various studies have confirmed that *BCL7A* is recurrently mutated in BL at frequencies of ~ 7% in coding regions and up to 43% when also considering non-coding regions, harboring mutational patterns consistent with aSHM [108, 138]. However, it is unclear if the coding or non-coding mutations of *BCL7A* are under positive selection in BL [135].

Importantly, some SWI/SNF genes are differentially mutated in the different subtypes of BL. For example, mutations in *BCL7A* are, together with those in *BCL6*, highly enriched in the endemic subtype of BL, which is associated with Epstein-Barr virus (EBV) infection, whereas mutations in *SMARCA4* are enriched in the sporadic subtype, which is usually EBV-negative [122, 138]. In addition, mutations in *ARID1A*, *SMARCA4,* and *BCL7A* are significantly depleted in human immunodeficiency virus (HIV)-associated BL [122].

#### Hodgkin lymphoma

HL has two major subtypes: classical HL (cHL) and nodular lymphocyte-predominant HL (NLPHL). Two SWI/SNF genes have been proposed as drivers of cHL based on their accumulation of mutations: *ARID1A* (mutation frequency ~ 9-26%) and *ACTB* (mutation frequency ~ 26%) [112, 139, 140]. Whereas mutations in *ARID1A* are mostly deleterious (e.g., nonsense, frameshift, or splice-altering), mutations in *ACTB* are usually missense. *ARID1A*-mutant cHLs have an increased burden of driver mutations, which may be associated with a higher sensitivity to PD-1 blockade [179]. On the other hand, mutations in *ACTB* may be associated with aberrant cytokinesis, which is thought to be a hallmark of cancerous HL cells [140]. Other SWI/SNF genes that may be recurrently mutated in cHL include *BCL7A* and *SMARCA4* [180, 181]. Furthermore, gain of chromosome 2p16.1, which contains *BCL11A*, is the most recurrent copy number alteration in cHL, affecting ~ 55% of the cases (*N* = 44) [127]. Finally, to our knowledge, no SWI/SNF mutations have been described in NLPHL, although only a couple dozen samples have been sequenced comprehensively so far [182, 183].

#### Other B-cell lymphomas

In primary mediastinal large B-cell lymphoma (PMBL), a wide variety of SWI/SNF genes are recurrently mutated, but discrepancies between studies are high [112, 141, 184]. Whereas one study reported that the top mutated SWI/SNF genes in PMBL are *ARID1A* (21%, *N* = 33) and *SMARCA4* (15%, *N* = 33) [112], another argued that only *ACTB* is mutated above the background mutation rate (33%, *N* = 95) [141]. Moreover, gains of chromosome 2p16.1 affecting *BCL11A* are frequent in PMBL [128, 129].

In mantle cell lymphoma (MCL), recurrent mutations have been observed in *SMARCA4* (mutation frequency ~ 6-10%), *ARID2*, *ARID1A*, and *ARID1B* (mutation frequency ~ 3-6% each) [108, 112, 142, 143]. Other SWI/SNF genes, such as *SMARCB1*, *SMARCD1*, *PBRM1*, and *BCL11B*, are mutated at lower frequencies [112].

In marginal zone lymphoma (MZL), recurrent mutations have been reported in *ARID1A* (7%) and *ARID1B* (4%) [112]. Interestingly, approximately half of the mutations in both genes are frameshift or nonsense. The mutation frequencies are consistently lower in meta-analyses than in individual studies, possibly because not all studies included in meta-analyses may have sequenced both genes [144, 145]. Other SWI/SNF genes recurrently mutated at lower frequencies are *SMARCA4*, *SMARCB1*, and *PBRM1*.

SWI/SNF genes are also frequently altered in lymphoplasmacytic lymphoma (LPL), including its most common subtype, known as Waldenström Macroglobulinemia (WM), and its precursor form, known as IgM monoclonal gammopathy of undetermined significance. In particular, *ARID1A* harbors recurrent inactivating mutations in ~ 5-17% of WM patients, suffering biallelic inactivation in some cases [112, 147, 148]. Furthermore, *ARID1B* is deleted in 50% of WMs [148].

### T- and NK-cell lymphomas

The most common mature T cell neoplasm is peripheral T cell lymphoma, not otherwise specified (PTCL, NOS). In various PTCL, NOS cohorts, recurrent point mutations have been reported in *ARID1A*, *ARID2*, *ARID1B*, *SMARCA2*, and *SMARCA4* [110, 112, 149–151]. Importantly, most mutations in *ARID1A* and *ARID2* are truncating (e.g., nonsense or frameshift), and each gene may be deleted in ~ 5% PTCLs, NOS [110, 149].

Cutaneous T cell lymphomas (CTCLs) include mycosis fungoides and Sézary syndrome. In CTCLs, somatic copy number alterations are an order of magnitude more frequent than point mutations [152–154]. Importantly, *ARID1A* is among the top altered genes in CTCLs, especially in Sézary syndrome [112, 152, 153]. In particular, the frequencies of *ARID1A* deletions in various CTCL cohorts were 28% (*N* = 25), 32% (*N* = 37), 58% (*N* = 40), and 55% (*N* = 94) [152–155]. Furthermore, point mutations in *ARID1A* have been identified in ~ 8% CTCLs (combined *N* = 679), and about half of them are truncating [112, 152–157]. Interestingly, most *ARID1A* mutations and deletions are heterozygous. Although it has been proposed that the remaining wild-type allele may be silenced by epigenetic mechanisms, leading to no ARID1A protein expression, further data are needed to confirm this hypothesis [152]. Other SWI/SNF genes recurrently altered in CTCLs are *SMARCE1* and *SMARCD2* (each amplified in 20% Sézary syndrome patients, *N* = 25), *ARID2* (deleted in 8% Sézary syndrome patients, *N* = 25), and *SMARCB1* (point mutation frequency ~ 3%, combined *N* = 433) [112, 152–154, 156]. Finally, one study reported that *BCL7A* is recurrently deleted in mycosis fungoides (frequency = 44%, *N* = 16) [158], but this finding was not reproduced by a later study (*N* = 22) [185].

In hepatosplenic T cell lymphoma (HSTCL), chromatin remodeling genes are mutated in over 60% of the patients [109]. In particular, *ARID1B* is mutated in 18% HSTCLs, and *SMARCA2* is mutated in 10% HSTCLs (*N* = 68). However, over a third of the reported *ARID1B* mutations are synonymous, and therefore their contribution to oncogenesis is unclear.

SWI/SNF mutations are rare (≤5-6%) in other types of T cell lymphomas, including angioimmunoblastic T cell lymphoma [186–190], extranodal NK/T cell lymphoma [159–163], monomorphic epitheliotropic intestinal T cell lymphoma [191–193], and anaplastic large-cell lymphoma (ALCL) [112, 194–197]. The mutations, if any, mostly affect *ARID1A,* and many are truncating. Interestingly, one ALCL case suffered a fusion between *ARID1A* and *EPB41* [194]. Finally, non-synonymous mutations in *BCL11B* may affect 12% of enteropathy-associated T-cell lymphomas [164].

### Multiple myeloma

Although comprehensive catalogs of MM driver genes are currently available, SWI/SNF genes do not seem to be mutated at high recurrence in their coding sequences [198, 199]. On the other hand, mutations in the 5’-UTR of *BCL7A* affect up to 76% of MM patients [123, 199, 200]. *BCL7A* is a tumor suppressor gene that is downregulated in MM compared to normal plasma cells, but it is unclear whether the non-coding mutations observed in MM affect BCL7A expression and whether the mutations are under selection [123]. The patterns of mutations in *BCL7A* in MM may be consistent with AID activity, whose mutational signature in MM is mostly found in non-coding regions [201]. Future research should explore whether the non-coding mutations in *BCL7A* in MM are a mere sign of past AID activity or whether they are under selection.

### B- and T-cell leukemias

Acute lymphoblastic leukemia (ALL) can affect the hematopoietic precursors of both B and T cells. In B-ALL, although SWI/SNF alterations are rare (≤5%), recent statistically powerful studies have predicted some SWI/SNF genes as putative drivers. In particular, Ma et al. reported that *ARID2* (11/218 mutant patients, 5%) and *ACTB* (1.4%) may be B-ALL drivers [165]. On the other hand, Brady et al. identified as candidate B-ALL drivers *ARID2* (2.5% genetic alteration frequency, *N* = 1428), *ARID1A* (< 1%), *ACTB* (< 1%), and *BICRAL* (< 1%), all of which harbored mostly truncating mutations [166]. In T-ALL, *BCL11B* is a well-known driver gene that is mutated in 8-10% of the cases (combined *N* = 1161) [98, 112, 165–167]. The mutations are mostly heterozygous and inactivating (e.g., deletions and missense mutations that are predicted to disrupt its DNA-binding domain), suggesting that *BCL11B* may be a haploinsufficient tumor suppressor in T-ALL. Furthermore, recurrent genomic rearrangements in ~ 2-3% of T-ALLs place *TLX3* under the control of *BCL11B* enhancers, which may in turn inactivate *BCL11B* expression. Other putative driver genes in T-ALL include *ARID1A* (~ 3%) and *SMARCA4* (~ 3%), the latter of which is mutated in 14% of *TLX3*-rearranged T-ALLs [165, 166].

In chronic lymphocytic leukemia (CLL), mutations in SWI/SNF genes are rare, affecting < 6% of the patients [112, 113]. The most recurrently mutated SWI/SNF gene is *ARID1A*, which is mutated in ~ 0.9-1.6% of CLLs (*N* = 1643). Other recurrently mutated SWI/SNF genes in CLL are *ARID1B* (1.3%) and *PBRM1* (0.9%) [112]. Importantly, over 80% of the *ARID1A* mutations and over 60% of the *ARID1B* mutations in CLL are nonsense or frameshift. Finally, recurrent loss of chromosome 22q11 (which encompasses *SMARCB1*) has been reported in up to 55% of T-cell prolymphocytic leukemias (T-PLL) (combined *N* = 34), but *SMARCB1* may not be significantly downregulated in the affected cases [168, 169].

### Myeloid malignancies

In MDS, *ARID2* has been identified as a putative driver by some studies (reviewed by [202]). Although the frequency of somatic point mutations in *ARID2* is relatively low (~ 1-2%, combined *N* = 898), the mutations are mostly truncating, and they are thought to be early events [112, 171]. Moreover, *ARID2* is deleted in an additional ~ 1% MDSs [171]. Mutations and deletions in *ARID2* in MDS may co-occur and act synergistically with those in components of the Polycomb Repressive Complex 2 (PRC2), especially *EZH2* and *JARID2* [171]. Overall, *ARID2*-deficient MDS has been proposed as a distinct entity with unique genetic and phenotypical features [171]. Besides *ARID2*, no other SWI/SNF gene is generally considered to be a driver of MDSs, although rare mutations in *ARID1A* and *ARID1B* have also been reported (mutation frequency ~ 0.5% each) [112]. In stark contrast to previous studies, Yao et al. reported that SWI/SNF genes are mutated in 17.8% of MDSs in a single-center cohort (*N* = 118) [172]. The top 3 mutated SWI/SNF genes were *ARID1A*, *ARID2*, and *ARID1B*. The source of the large discrepancies between Yao et al’s report and previous studies is unclear.

In other myeloid malignancies, mutations in SWI/SNF genes seem to be extremely rare (< 1%), and, if present, they mostly affect *ARID1A*. This is the case for AML [51], CML [203], and myeloproliferative neoplasms such as juvenile myelomonocytic leukemia [204, 205], chronic myelomonocytic leukemia [206], chronic neutrophilic leukemia [207], chronic eosinophilic leukemia [208], and others [112, 209–213]. However, there are notable exceptions. In APL, recurrent point mutations (mostly truncating) have been found in *ARID1A* (5%, *N* = 165) and *ARID1B* (3%) [84]. Interestingly, *ARID1B* mutations were enriched in relapsed APL (12%, *N* = 77) compared to newly diagnosed APL. In addition, recurrent deletions of *SMARCB1* in CML patients have been reported at frequencies ranging from 30% (*N* = 46) [91] to 80% (*N* = 20) [170].

### Other hematological malignancies

About one third of ALALs are characterized by genomic alterations that cause aberrant activation of *BCL11B* [99, 100]. There are two main mechanisms by which this activation occurs: (i) translocations that place *BCL11B* under the control of enhancers that are active in HSPCs; or (ii) de novo generation of a superenhancer by tandem amplification of an enhancer located downstream of *BCL11B* [99, 100]. Interestingly, the most frequent event places *BCL11B* under regulatory elements located upstream of *ARID1B*. Furthermore, aberrant activation of *BCL11B* strongly co-occurs with *FLT3* alterations.

In blastic plasmacytoid dendritic cell neoplasm (BPDCN), mutations (mostly loss-of-function) and deletions in *ARID1A* seem to be recurrent, although cohort sizes have been low (8 altered patients, combined *N* = 70) [111, 112, 173–176]. Importantly, loss-of-function *ARID1A* mutations have been inferred to be early events in at least some BPDCNs [176]. Another WES study in BPDCN, which mostly lacked matched normal samples, found 15/47 (32%) cases mutated in *ARID1A* [214]. Most of the mutations in *ARID1A* were missense. Furthermore, the authors defined two molecular subtypes of BPDCN, one of which was enriched in mutations in *ARID2, SMARCA4*, and *PBRM1* [214].

SWI/SNF genes are also altered in extremely rare hematological malignancies, some of which only have one reported case. For example, a recent case report described a highly aggressive pediatric hematopoietic malignancy that was associated with biallelic loss of *SMARCB1*, a phenomenon that is normally associated with rhabdoid tumors [215].

Finally, recurrent SWI/SNF mutations have been identified at low frequencies in other hematological malignancies, albeit mostly in limited cohorts lacking matched normal samples. These include *ARID1B*, *ARID2*, *BCL11B*, and *SMARCA2* mutations in post-transplant lymphoproliferative disorders [189, 216], *ARID1A* mutations in primary histiocytic sarcoma [217], and *ARID1A* mutations in aggressive Langerhans cell histiocytosis [218].

## Epigenetic and other expression alterations

Epigenetic alterations are defined as heritable modifications of phenotype that do not affect the DNA genomic sequence itself, and they are important regulators of gene expression [219]. In hematological [92] and non-hematological [117] malignancies, expression alteration of SWI/SNF subunits attributable to epigenetic silencing or other mechanisms has been reported (Table 4).

**Table 4:** Altered expression (Tumor/Non-tumor) of SWI/SNF members in hematological malignancies

SWI/SNF subunit	Tumor type	Expression (T/N)	Mechanism(s)	Reference
ARID1A	AML	Down	Unknown	[89]
ARID1B	CLL	Down	miRNA regulation	[220]
BCL7A	MM	Down	5’UTR mutations	[123]
	CTCL	Down	Hypermethylation	[221]
BCL11A	CLL, NHL, HL, ALL, AML, CML, NKTL	Up	Chromosomal translocation, amplification	[93, 96, 222–226]
BCL11B	ALAL	Up	Chromosomal translocation, enhancer amplification	[99]
SMARCA4	Leukemia & lymphoma cell lines	Down	miRNA regulation	[227, 228]
SMARCB1	AML	Down	Hypermethylation	[92]
	CTCL (SS)	Down	Unknown	[229]

*ALAL* acute leukemia of ambiguous lineage, *ALL* acute lymphoid leukemia, *AML* acute myeloid leukemia, *CLL* chronic lymphocytic leukemia, *CML* chronic myeloid leukemia, *CTCL* cutaneous T-cell lymphoma, *HL* Hodgkin lymphoma, *MM* multiple myeloma, *NHL* non-Hodgkin lymphoma, *NKTL* NK/T-cell lymphoma, *SS* Sézary syndrome

One of the most widely studied mechanisms of epigenetic regulation is promoter hypermethylation. For example, *SMARCB1* is downregulated in AML because of repressive methylation of the CpG islands in its promoter, compared to non-tumor hematopoietic cells [92]. Moreover, in CTCL, one study found that the promoter region of *BCL7A* was hypermethylated, and its expression was successfully restored after treatment with a demethylating agent (decitabine) [221]. However, to our knowledge, little evidence has been found of hypermethylation affecting other SWI/SNF genes in hematological malignancies.

Chromosomal translocations and genomic amplifications can also be a source for expression alterations, such as the ones affecting *BCL11A*. However, as stated in previous sections, it is unclear if *BCL11A* is a specific target of these genomic alterations, and, in fact, these amplifications are often not associated with increases in *BCL11A* mRNA expression [8, 129, 131]. Regardless, many studies have shown that BCL11A is upregulated in several B cell malignancies (CLL, NHL, Hodgkin disease), as well as in ALL, AML, CML, and natural killer/T cell lymphoma (NKTL) [93, 96, 222–226]. Furthermore, aberrant allele-specific expression of *BCL11B* is a defining feature of a subgroup of ALALs [99].

Finally, another mechanism of gene expression regulation are microRNAs (miRNAs). MiRNAs are small, ~ 18-25 nucleotides long, highly conserved non-coding RNAs that regulate gene expression by binding to target mRNAs, usually impeding their translation and, thus, downregulating their expression [230]. The regulation of SWI/SNF complex subunits by miRNAs has been studied in different tumors. For instance, *SMARCA4* has been found to have two alternative 3’UTRs in lung tissue containing active binding sites for miR-155, miR-199, and miR-101 [231]. Because the activity of miRNAs is frequently tissue-specific, it is unknown whether this regulation could be extended to hematological malignancies. At least, that seems to be the case for the regulation of *SMARCA4* by miR-155 in some hematological malignancies [227, 228]. In another study in CLL patients, a series of upregulated miRNAs (miR-22, miR-34a, miR-146b, and miR-181b) were found to be responsible for the downregulation of *ARID1B* [220].

## Clinical implications of hematological malignancies with altered SWI/SNF complexes

**Fig. 3:**
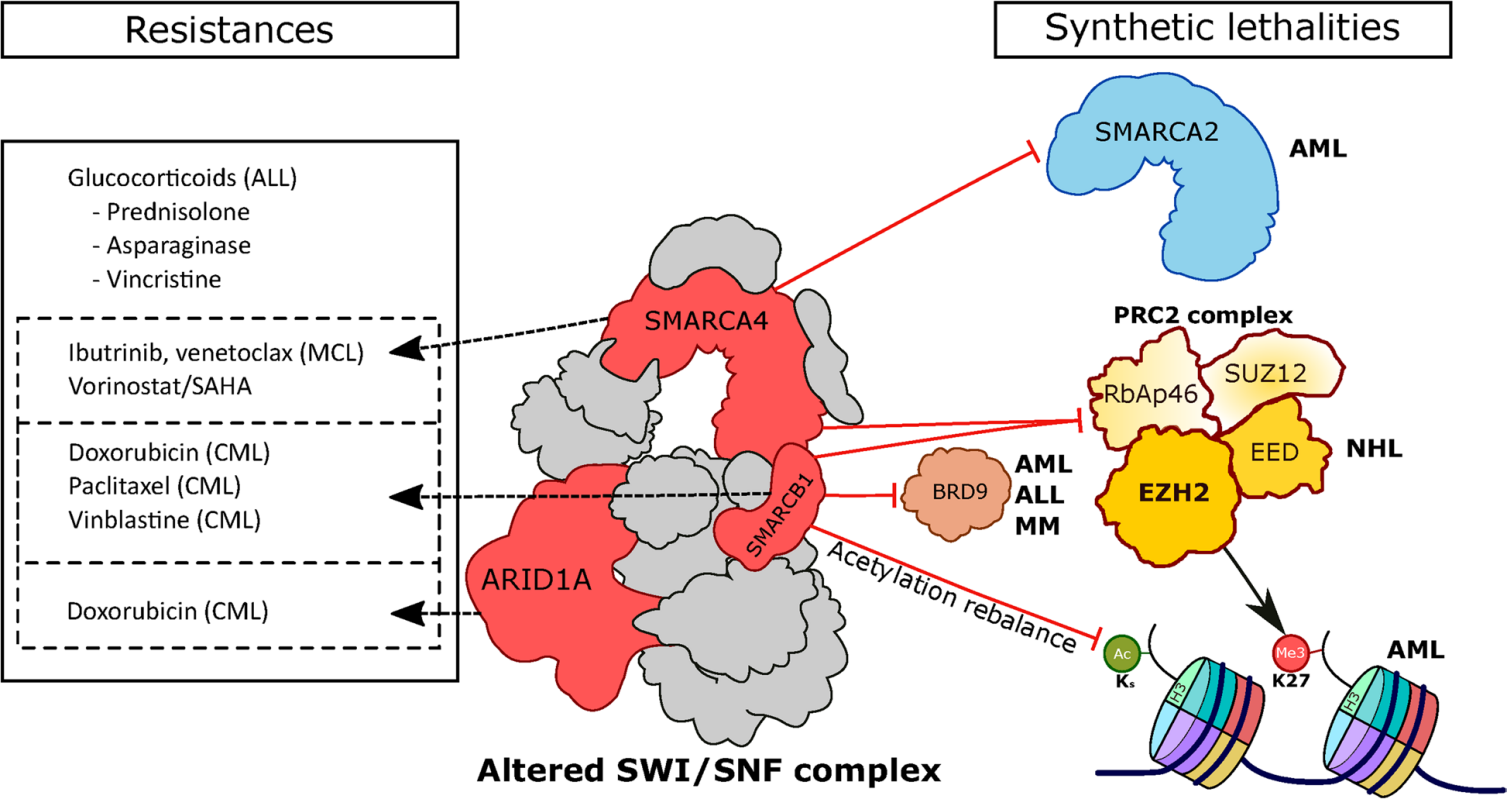
Therapeutic opportunities and resistances generated by altered SWI/SNF complexes in hematological malignancies. Altered subunits (depicted in red) create dependencies on other subunits or pathways/activities that can be exploited (blunt red arrows). Aberrant SWI/SNF complexes confer resistance to routinely used antineoplastic drugs (black arrows). The rest of the subunits of the SWI/SNF complex are shown in grey. ALL: acute lymphoblastic leukemia; AML: acute myeloid leukemia; CML: chronic myeloid leukemia; MCL: mantle cell lymphoma; MM: multiple myeloma; NHL: non-Hodgkin lymphoma; PRC2: polycomb repressive complex 2

The recurrent alteration of SWI/SNF complexes during cancer development not only shows the biological relevance of these chromatin remodelers in hematological malignancies, but also highlights their clinical potential. Growing evidence has demonstrated that alterations in SWI/SNF genes result in vulnerabilities and drug resistance phenomena in hematological malignancies, among other cancers (Fig. 3). Some of these susceptibilities are subunit and/or cell-type specific, while others could be more widely applicable.

### Altered SWI/SNF complex and drug resistance

Increasing evidence has shown that mutations in SWI/SNF complex subunits confer resistance to several antineoplastic agents routinely used for the treatment of hematological malignancies, including ibrutinib, venetoclax, doxorubicin, paclitaxel or vinblastine. Specifically, in MCL, an upregulation of the pro-survival gene *BCL2L1* (Bcl-xL) has been observed when *SMARCA4* harbors loss-of-function mutations, which results in primary resistance to ibrutinib and venetoclax treatments or, eventually, in relapse after dual exposure to these agents [232]. Likewise, in the haploid CML cell line HAP1, depletion of SMARCB1 or ARID1A increased resistance to doxorubicin, and depletion of SMARCB1 also increased resistance to paclitaxel and vinblastine [233]. In the case of SMARCB1, the resistance was created through the enhancement of the expression of ABCB1 (ATP binding cassette subfamily B member 1), an ATP-dependent promiscuous drug efflux pump, which mediates a well-known mechanism of resistance to several chemotherapeutic drugs [234]. In this scenario, removing SMARCA4 could reverse the effects of the loss of SMARCB1, including ABCB1 overexpression and doxorubicin resistance. Consequently, designing clinical approaches that aim to target these pathogenic residual SWI/SNF complexes may boost sensitivity to chemotherapeutics. Moreover, SWI/SNF complexes are also required for glucocorticoid-dependent transcription. Glucocorticoids, such as prednisolone, asparaginase, or vincristine, are regularly used for the treatment of ALL, and resistance to glucocorticoids has been defined as an important adverse prognostic factor in newly diagnosed ALL patients. Increased expression of several SWI/SNF subunits, including SMARCA4, ARID1A, SMARCB1, and SMARCC2 has been associated with prednisolone sensitivity, whereas the lack of expression correlates with resistance to prednisolone treatment in ALL [235].

Finally, the FDA has approved Vorinostat for the treatment of cutaneous T-cell lymphoma. This drug, also known as SAHA, inhibits all zinc-related HDACs [236], being the first HDAC inhibitor applied to clinical studies. When applied as a single agent, it has exhibited limited results [237], although preclinical studies of a DLBCL therapy combining SAHA and EZH2 inhibitors have shown a clear synergistic effect [238]. In addition, it has been described that lung tumoral cells lacking the SWI/SNF-subunit SMARCA4 are refractory to SAHA treatment [239].

### Synthetic lethality-based therapeutic approaches for altered SWI/SNF complex subunits in hematological malignancies

Mutations in SWI/SNF genes often create dependencies either on genes encoding other SWI/SNF subunits or on other pathways or activities that may become therapeutic targets in this context. These phenomena point to a scenario where subunit mutations do not completely halt SWI/SNF activity, resulting in abnormal SWI/SNF complexes with an alternative residual activity that supports cancer progression [240, 241]. Synthetic lethality occurs when two genetic events (i.e., mutations) are combined, resulting in a loss of cell fitness. Several synthetic lethal relationships have been described in altered SWI/SNF-driven cancers whereby cell viability upon the loss of one subunit (e.g., SMARCA4, SMARCB1, ARID1A) uniquely relies on the presence of either other SWI/SNF paralog subunit or a downstream gene or cellular pathway [242]. One of the best-characterized examples of the synthetic lethality between subunit paralogs has been described in non-small cell lung carcinoma cell lines lacking SMARCA4, where SMARCA2 has been identified as the upmost synthetic lethal dependency necessary for cell proliferation [243].

#### Synthetic lethality between SWI/SNF complex subunits

In hematological malignancies, most of the SWI/SNF subunit-targeting therapies described so far are based on small inhibitors targeting either SMARCA4/SMARCA2 or BRD9. Given that either loss-of-function point mutations or inactivation of SMARCA4 have been outlined in several hematological malignancies, targeting its paralog (SMARCA2) might be a potential therapeutic alternative for the treatment of these cancers. Indeed, it has been shown that several hematopoietic cancer cell lines are exquisitely sensitive to dual SMARCA4/SMARCA2 catalytic inhibitors [244, 245]. A further study in AML revealed that this sensitivity is not driven entirely by SMARCA4 dependence, but also requires the concomitant deprivation of SMARCA2 activity [88]. Hence, SMARCA2/SMARCA4 inhibitors target recurrent leukemic transcriptional programs in cell lines, resulting in a wide range of phenotypic effects, including apoptosis and differentiation. This results in a reduction of tumor growth in an AML xenograft mouse model after the treatment, thus demonstrating the therapeutic potential of SWI/SNF inhibition. This therapeutic avenue can be streamlined with the recent development of proteolysis targeting chimera (PROTAC) degraders of SMARCA2 and SMARCA4 [246], and further optimization of a SMARCA2 selective, orally active VHL-based degrader [247]. Indeed, the clinical use of PROTACs could be of great interest, given the success of several clinical trials where PROTACs are being applied to inhibit key proteins in hematological malignancies, other than SWI/SNF. These clinical trials include targets such as IRAK4 in DLBCL, BTK in B-cell malignancies, IKZF1/3 in MM and CML, and GSPT1 in AML [248]. Likewise, BRD9, a key component of the ncBAF complex, has been proposed as a therapeutic target in a group of leukemias. Particularly in AML, small-molecule inhibitors of BRD9, including BI-7273 and I-BRD9, regulate the cell proliferation rate [102]. Moreover, with the help of PROTACs developed to target BRD9 (dBRD9 and VZ185), it has been possible to verify that anti-cancer activity against AML might be achieved just by blocking the BRD9 bromodomain. However, in those cancers where SMARCB1 is mutated the blockage of the bromodomain is not sufficient, requiring a complete degradation of BRD9, hinting to a need for structural disruption of ncBAF [249–251]. More recently, Weisberg and collaborators [101] analysed the dependency on BRD9 in a variety of hematological cancers, including MM, ALL and AML, using novel small molecule inhibitors (EA-89-YM35), degraders (QA-68-ZU81), and RNA interference. They found that after depletion of the BRD9 protein, apoptosis was prominently triggered in ALL and MM, whereas AML cells exhibited terminal differentiation. In addition, these authors observed that the effects of a plethora of chemotherapeutic compounds and targeted therapies against MM, AML and ALL were enhanced upon BRD9 degradation. These findings point to a novel strategy for ALL and MM through the targeting of BRD9, either alone or combined with other compounds.

#### Synthetic lethality involving non-SWI/SNF complex subunits

Regarding synthetic lethal dependencies displayed by mutant SWI/SNF complexes and other proteins involved in different cellular pathways, Polycomb Repressor Complexes (PRCs) stand out as one of the best-studied examples. PRCs have opposing gene-regulatory activities to those of SWI/SNF [252]. Specifically, the recruitment of SWI/SNF complexes results in a displacement of both PRC1 and PRC2 that is abolished when SMARCB1 is mutated [253]. Thus, the synthetic lethal relationship between PRC2 inhibition and SMARCB1 loss is one of the most actionable dependencies that can be currently targeted with inhibitory compounds against the core subunits of PRC2, EZH2 and EED [254, 255]. Among the different EZH2 inhibitors that have been developed, tazemetostat, also known as EPZ-6438, has shown promising preclinical results [237, 256–258]. This low molecular weight compound is a highly specific competitive inhibitor of the cofactor S-adenosyl methionine (SAM) with limited effects in other lysine methyl transferases, including EZH1. This compound reduces trimethylation marks given that SAM is required for H3K27 methylation by EZH2 [257]. Noticeably, there is an active phase II clinical trial (NCT03213665-MATCH) aiming to determine the performance of tazemetostat in pediatric NHL patients harboring *SMARCB1* or *SMARCA4* gene mutations. Interestingly, the synthetic lethality displayed between SWI/SNF and PRC2 is being assessed not only in hematological malignancies, but in solid tumors as well. Indeed, a phase II clinical trial (NCT05023655) is recruiting patients to analyze the clinical benefit of tazemetostat in *ARID1A*-mutant solid tumors.

Given that SWI/SNF complexes contribute to the regulation of enhancer function by facilitating the acetylation of H3K27, defective complexes may alter H3K27 acetylation patterns and unleash or support cancerous transcriptional programs. Therefore, further investigation of reagents that modify histone acetylation levels might be of interest regarding hematological malignancies with abnormal SWI/SNF complexes [259]. In this line, it has been shown that loss of SMARCB1 increased recruitment of an endogenous, residual, nuclear SWI/SNF complex and associated histone acetyltransferases (HATs) to target loci, thereby promoting H3K27Ac and thus gene expression that, through Rac activation, enhanced AML cell migration and survival. This finding highlights the tumor suppressor role of SMARCB1 and illustrates the function of a residual SWI/SNF complex in maintaining an oncogenic gene expression program in AML [92].

## Conclusion and perspectives

SWI/SNF complexes are ubiquitous epigenetic regulators that play major roles in normal and aberrant hematopoiesis. Indeed, a wide variety of hematological malignancies harbor recurrent alterations in SWI/SNF genes, and the clinical implications of such alterations are just starting to be explored. Recent studies on SWI/SNF-targeting chemotherapeutic agents, as well as on the role of SWI/SNF alterations in drug resistance and the creation of targetable synthetic lethalities in SWI/SNF-defective tumors, are opening new paths for improving cancer treatment that hold a promising future. On the other hand, mechanistic knowledge on the role of SWI/SNF in hematological malignancies is largely limited, and improving this knowledge may unlock novel therapeutic opportunities.

In-depth mechanistic studies of SWI/SNF function and of the consequences of SWI/SNF alterations are mostly lacking in hematological contexts, especially outside of AML. Most knowledge is relatively recent, as advanced epigenomic techniques are necessary to unveil the complex mechanisms of action of SWI/SNF. Importantly, one major question is why similar SWI/SNF alterations are found across such a wide range of hematological and non-hematological cancers. The involvement of SWI/SNF complexes in DNA repair and genome stability may help to explain this observation. In addition, recent work has revealed that SWI/SNF targets lineage-specific regulatory elements across the genome, and that at least some loss-of-function alterations in SWI/SNF genes affect their accessibility. However, most of this work has been performed either in non-hematological models or in AML, which is rarely mutated in SWI/SNF genes. Therefore, future research should explore the role of wild type and mutant SWI/SNF on the accessibility of lineage-specific loci with special emphasis on hematological malignancies that have a high mutation rate of SWI/SNF genes, such as DLBCL.

Another layer of complexity is the fact that the subunit composition of SWI/SNF is dynamic, which has critical implications in mechanistic, phenotypical, and clinical studies. Specifically, when a SWI/SNF subunit is inactivated by either mutation, silencing, or drug treatment, residual SWI/SNF complexes may remain functional, and they may even gain new functions that can be oncogenic, as is the case of SMARCB1-deficient AML. In turn, these phenomena can generate synthetic lethality relationships that can be exploited therapeutically. In this context, future studies should explore further synthetic lethality relationships in SWI/SNF-mutant hematological cancers, which may reveal novel therapeutic opportunities.

Furthermore, novel lines of research could aim to therapeutically rectify alterations in the expression of SWI/SNF genes in hematological malignancies. For example, demethylating agents may restore the expression of hypermethylated SWI/SNF genes to deliver tumor suppressor activity, as has been shown in preclinical studies. Moreover, future studies could explore the therapeutic potential of targeting SWI/SNF subunits that are overexpressed in cancer, such as BCL11A or BCL11B, using approaches such as RNA interference or PROTACs.

Overall, in our view, ongoing preclinical and clinical studies are only scratching the surface of the clinical potential of targeting SWI/SNF or exploiting its synthetic lethality relationships in hematological malignancies, and we expect that future research will reveal new options for improving the treatment of such a diverse group of cancers.

## Abbreviations

ABCB1: ATP binding cassette subfamily B member 1
AID: Activation-induced cytidine deaminase
ALAL: Acute leukemia of ambiguous lineage
ALCL: Anaplastic large-cell lymphoma
ALL: Acute lymphoblastic leukemia
AML: Acute myeloid leukemia
APL: Acute promyelocytic leukemia
ARID: AT-rich interaction domain
aSHM: Aberrant somatic hypermutation
ATRA: All-trans retinoic acid
BAF: BRG1/BRM-associated factor
BCL7A: B-cell CLL/lymphoma 7 protein family member A
BL: Burkitt lymphoma
BPDCN: Blastic plasmacytoid dendritic cell neoplasm
cHL: Classical HL
CLL: Chronic lymphocytic leukemia
CML: Chronic myeloid leukemia
CTCLs: Cutaneous T cell lymphomas
DLBCL: Diffuse large B-cell lymphoma
EBAFb: ENL-associated BAF-containing BAF250b
EBV: Epstein-Barr virus
FL: Follicular lymphoma
GCB: Germinal center B cell-like
GENIE: Genomics, Evidence, Neoplasia, Information, Exchange
HAT: Histone acetyltransferase
HIV: Human immunodeficiency virus
HL: Hodgkin lymphoma
HSC: Hematopoietic stem cell
HSPC: Hematopoietic stem and progenitor cell
HSTCL: Hepatosplenic T cell lymphoma
ICGC: International Cancer Genome Consortium
LPL: Lymphoplasmacytic lymphoma
MCL: Mantle cell lymphoma
MDR1: Multidrug resistant
MDS: Myelodysplastic syndrome
MM: Multiple myeloma
MPN: Myeloproliferative neoplasm
MZL: Marginal zone lymphoma
ncBAF: Non-canonical BAF
NHL: Non-hodgkin lymphoma
NK: Natural killer
NKTL: Natural killer/T cell lymphoma
NLPHL: Nodular lymphocyte-predominant HL
PBAF: Polybromo-associated BAF
PgP: P-glycoprotein pump
PLL: Prolymphocytic leukemia
PMBL: Primary mediastinal B-cell lymphoma
PRC: Polycomb repressor complex
PROTAC: Proteolysis targeting chimera
PTCL, NOS: Peripheral T cell lymphoma, not otherwise specified
SS: Sézary syndrome
SWI/SNF: Switch/Sucrose Non-Fermentable
UTR: Untranslated region
WES: Whole-exome sequencing
WGS: Whole-genome sequencing
WM: Waldenström macroglobulinemia
